# Influence of revised public health standards on health equity action: a qualitative study in Ontario, Canada

**DOI:** 10.1186/s12939-017-0677-9

**Published:** 2017-10-27

**Authors:** Nadha Hassen, Ingrid Tyler, Heather Manson

**Affiliations:** 1 The Upstream Lab, Centre for Urban Health Solutions, Li Ka Shing Knowledge Institute, St. Michael’s Hospital, 30 Bond Street, Toronto, ON M5B 1W8 Canada; 2 Fraser Health Authority, Suite 400, 13450 – 102nd Avenue, Surrey, BC V3T 0H1 Canada; 3 Public Health Ontario (Ontario Agency for Health Protection and Promotion), Suite 300, 480 University Avenue, Toronto, ON M5G 1V2 Canada; 4 Dalla Lana School of Public Health, University of Toronto, 155 College Street, 6th floor, Toronto, ON M5T 3M7 Canada; 5 School of Public Health and Health Systems, University of Waterloo, Waterloo, ON N2L 3G1 Canada

**Keywords:** Health equity, Social determinants of health, Priority populations, Ontario Public Health Standards

## Abstract

**Background:**

In 2008, a revised set of public health standards was released in the province of Ontario, Canada. The updated Ontario Public Health Standards (OPHS) introduced a new policy mandate that required local public health units (PHUs) to identify “priority populations” for public health programs and services. The aim of this study was to understand how this Priority Populations Mandate (PPM) facilitated or hindered action on health equity or the social determinants of health through PHUs in Ontario.

**Methods:**

This study used two sets of qualitative data that were part of a larger study. The first set of data was 16 semi-structured key informant interviews with policymakers involved in developing the OPHS and public health practitioners. The second set of data was the qualitative component of a role-based survey sent out to all the 36 PHUs in Ontario. Thematic content analysis was conducted to iteratively develop themes to answer the research question.

**Results:**

We identified six factors that both facilitated and hindered action on health equity and social determinants of health action in the province resulting from the OPHS and PPM. These six factors were grouped into three categories or themes: OPHS policy attributes (1. introducing new terminology, 2. allowing flexibility in implementation and 3. ensuring evidence-informed decision-making), health sector context into which the PPM was introduced (4. different understandings of health equity and 5. variability in existing partnerships) and implementation by PHUs (6. requirement to address the PPM).

**Conclusions:**

Although the revised OPHS and the PPM facilitated action on health equity and the social determinants of health, on the whole, this objective could have been better met. The mandate within the OPHS could have been strengthened with respect to promoting action on health equity and the social determinants of health through more clearly defined terminology, conveying a guiding health equity vision and uniting different PHU approaches to addressing health equity.

## Background

Systemic health inequities, which are differences in health that are “unnecessary, avoidable and considered unfair and unjust” [1], are a prominent problem in Canada that require attention and action [2, 3]. In recent years, health equity has increasingly been seen as a goal of public health systems in Canada, with a number of initiatives arising to assist in addressing this issue across the health sector [2, 4, 5]. In Canada, health is provincially regulated and in the province of Ontario, the Ministry of Health and Long-Term Care (MOHLTC) oversees policies and direction of health care and public health. The MOHLTC sets out the minimal standards for public health programs and services in Ontario through the Ontario Public Health Standards (OPHS). There are 36 local Public Health Units (PHUs) within the province of Ontario that provide health promotion, protection and disease prevention programs to distinct geographic regions. Each PHU is an independent unit that reports to a Board of Health (BOH), which is required to comply with the OPHS [6]. The OPHS was revised in 2008 by the MOHLTC to replace the previous 1997 Mandatory Health Programs and Services Guidelines (MHPSG) [6]. As summarized by Pinto et al. [3], the revised public health standards had to be: 1) revenue- neutral 2) focused on evidence 3) focused on short-and long-term outcomes and performance and 4) less prescriptive to provide PHUs with the ability to tailor public health interventions to local needs.

The revised OPHS addressed health equity though introducing the term ‘priority populations’ and this new language was used predominantly throughout the 2008 OPHS [6] (p.1). The OPHS mandated that PHUs “assess the needs of the local population, including the identification of populations at risk, *to determine those groups that would benefit most from public health programs and services (i.e., priority populations)*” [6] (p.24). As the standards required local PHUs to “consider the determinants of health when identifying priority populations”, the link to health equity was inferred [6] (p.24), [4, 7, 8]. This mandate to identify priority populations, which we have called the Priority Population Mandate (PPM), was seen as enabling of action on health equity or the social determinants of health (SDOH) through the PHUs. However, with the revised public health standards, Pinto et al. [3] states that the term ‘priority populations’ was used as a proxy for health equity and that accordingly, the OPHS has a “lack of specificity” on health equity [3] (p.8). Throughout this paper, we use Priority Populations Mandate (PPM) to refer to the fact that the OPHS policy required PHUs to identify and take action on ‘priority populations’. Thus, the PPM is one component of the OPHS policy. To support implementation of the PPM within PHUs in Ontario, we undertook the Priority Populations Project [9], which had the following objectives:

- To clarify what priority populations are
- To clarify why priority populations should be identified
- To provide support on how to identify priority populations

This study is a sub-analysis of that larger project. This work builds on the paper by Pinto et al. [3] to understand how the OPHS facilitated or hindered health equity practice in the field [2]. The purpose of this study was to understand how the Priority Populations Mandate facilitated action on health equity (HE) or the social determinants of health (SDOH)^1^ through the Ontario Public Health Units (PHUs).

## Methods

Methods of the Priority Populations Project are provided in more detail in the Priority Populations Project Technical Report [9]. The project was approved by the Public Health Ontario (PHO) Research Ethics Board and used an Integrated Knowledge Translation strategy through collaboration with knowledge users, the alPHa-OPHA Health Equity Working Group. This sub-study draws on two of the three sets of data, namely the key informant interviews and the role-based survey, to answer the specific research question and does not triangulate data.

The first set of data was 16 semi-structured key informant interviews with policymakers involved in developing the OPHS and public health practitioners. They were identified through snowball sampling. Our aim was to gain an understanding of how the term ‘priority populations’ was intended to be used as written in the OPHS, as well as how the term is being applied by public health practitioners in the field. Since some practitioners who were implementing the mandate in the field were also involved in developing the OPHS, the 16 key informants fell into three key groups:

1. MOHLTC staff who were involved in the development of the OPHS (i.e., policymakers) (*n* = 4)
2. Current practitioners who were on the Technical Review Committee and contributed to the development of the OPHS (*n* = 6)
3. Current practitioners who were not involved in the development of the OPHS (*n* = 6)

Each of the three groups had their own semi-structured interview guide (which can be found in the Technical Report) [9]. Informed consent was obtained and the interviews were conducted, recorded and transcribed in July and August 2013 by one interviewer to ensure consistency. The interviewer (NH) also recorded notes and impressions directly after each interview and referred to these notes through the analysis process.

The second set of data was the qualitative component of a role-based survey sent out to all the 36 PHUs in Ontario. The online survey was developed with FluidSurveys and required active consent to access the survey [10]. The anonymous survey was distributed through institutional and professional listservs.

The survey was completed in October and November of 2013 by Medical Officers of Health and Associate Medical Officers of Health (MOH/AMOHs), Social Determinants of Health (SDOH) Nurses and PHU Epidemiologists. Individuals were not asked to identify their PHUs. The survey included multiple choice questions and free-text options; however only the free-text components of the survey were included in this study.

For the purposes of this study, we (NH and IT) went back to the transcripts of the key informant interviews and the free-text fields of the surveys that had previously been identified as a part of the Priority Populations Project. From our previous analysis of the transcripts, we had identified key statements. These key statements took the form of sentences and encompassed broad ideas rather than small phrases or words that were out of context. As such, it was possible to recode these key statements keeping in mind our research question for the sub-analysis (i.e., “How did the Priority Populations Mandate facilitate health equity or social determinants of health (SDOH) practice in Ontario Public Health Units (PHUs)?” We applied thematic content analysis to these key statements and inductively identified repeating themes related to what elements of the PPM facilitated or hindered HE/SDOH practice in PHUs. We did not conduct member checking with this sub-analysis.

Based on the themes that arose, factors of the PPM were grouped into either an attribute of the OPHS policy, context into which the PPM was introduced or PHU implementation of the PPM in the field. For example, the following quote by a policymaker was coded as an OPHS policy attribute because it describes the policy itself: “what we were trying to do was give each health unit the flexibility of addressing the health needs, as [identified] by epidemiology, of their communities.” Data were further subcategorized and then considered from the perspective of understanding how the PPM facilitated or hindered action on HE/SDOH. The above statement was then coded a second time as a facilitator of HE/SDOH action. During data analysis, the research team consciously engaged in reflection and challenged personal assumptions [9].

## Results

**Fig. 1:**
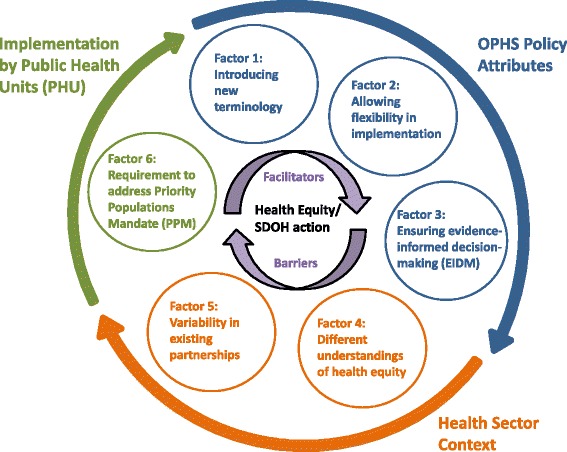
Conceptual model of influence of revised public health standards on HE/SDOH action. HE/SDOH action is depicted in the centre of the model as this was the focus of our research question. The six factors influencing HE/SDOH action are in the middle ring of the diagram and illustrate how each factor has both facilitators and barriers. Each factor is grouped within three categories or themes which are depicted on the outer ring and colour coded: 1) OPHS policy attributes 2) health sector context into which the PPM was introduced or 3) implementation by PHUs. The conceptual model is conceived of as a circle to highlight how health policies are implemented within a specific context and that implementation and practice should inform further policy

Based on our analysis, a broad understanding of how health equity is facilitated in Ontario emerged and this was depicted in a conceptual model (see Fig. 1). We identified six factors (see Table 1) which influenced how the PPM affected HE/SDOH action in Ontario. These six factors were grouped into three categories or themes: 1) OPHS policy attributes 2) health sector context into which the PPM was introduced and 3) implementation by the PHUs. The conceptual model highlighted the links between the overarching categories/themes and each of the six factors and how they contributed to HE/SDOH action in Ontario.

**Table 1:** Six Factors of the Priority Populations Mandate (PPM) that Influence HE/SDOH Action in Ontario

	PPM Factors Influencing HE/SDOH Action in Ontario
OPHS Policy Attributes	
Factor 1	Introducing new terminology
Factor 2	Allowing flexibility in implementation
Factor 3	Ensuring evidence-informed decision-making (EIDM)
Health Sector Context into which the PPM was introduced	
Factor 4	Different understandings of health equity
Factor 5	Variability in existing partnerships
Implementation by Public Health Units (PHUs)	
Factor 6	Requirement to address PPM

Each of these six factors had the potential to facilitate or inhibit HE/SDOH action in Ontario. The model depicted how these factors conceptually link to broader action on HE/SDOH while the specific ways in which the factors facilitated or inhibited are summarized in Tables 2 and 3 and discussed in more detail below (see Table 2: facilitators and Table 3: barriers).

**Table 2:** Aspects of the PPM Facilitating HE/SDOH Action

	Aspects of the PPM Facilitating HE/SDOH Action
OPHS Policy Attributes	The introduction of new language (i.e. term 'priority populations') opened up discussion
	The term ‘priority populations’ was seen as proactive
	The term ‘priority populations’ was perceived as value-neutral language
	Flexibility emphasized PHU role and autonomy in interpreting the PPM to fit their needs
	EIDM promoted objective conclusions due to business case of health equity / social justice
	PPM was perceived as organizing practice and directing resources through EIDM in an environment where justification for action on SDOH was challenging
Health Sector Context into which the PPM was introduced	PPM tried to overlay high-level population health thinking onto program delivery
	PPM tried to maintain balance between different schools of thought or ideological differences
	PPM promoted collaboration with different sectors
Implementation by Public Health Units (PHUs)	PPM was a catalyst that pushed PHUs to consider creative solutions and increased dialogue at local level
	PPM helped to counter negative perceptions that the health equity/ social justice approach had from a conservative viewpoint
	PPM made a connection between SDOH and health equity
	PPM assisted PHUs with making decisions in a tight funding environment
	PPM focused the work being done by PHUs, and spurred on and encouraged new work
	PPM drew attention of those PHUs who hadn’t been as engaged due to capacity issues, and increased mobilization
	PPM raised awareness of the need for HE capacity building within PHUs
	PPM identified opportunities for PHU partnerships; health equity work may be enhanced by sharing resources between PHUs
	PPM helped PHUs “do what they need to” and facilitated existing action

**Table 3:** Aspects of the PPM Inhibiting HE/SDOH Action

	Aspects of the PPM Inhibiting HE/SDOH Action
OPHS Policy Attributes	Introduction of new language was poorly defined and may have hindered progress
	New term caused confusion
	The issue of prioritizing populations created the concept of inherent ranking of populations
	Lack of evaluation, accountability and reporting mechanisms of the PPM meant there was no formal evaluation
	Priority populations identified through a ‘burden of disease first’ approach took away from HE/SDOH action
	EIDM required proof that a SDOH was causing a negative health outcome, which hindered progress on HE/SDOH action due to lack of available published evidence in some areas
	Data hindered HE/SDOH action because it highlighted data gaps which people found to be insurmountable
	The OPHS de-emphasized social justice and advocacy as some policymakers didn’t think HE/SDOH was linked to PPM
	Health equity was not crucial in PPM. That is, although health equity is seen as a part of the PPM, it is not the most important outcome
Health Sector Context into which the PPM was introduced	Different understandings of health equity caused confusion across professionals and health units, and talking at cross-purposes
	There was a need to collaborate with other sectors because issues may often be identified that are beyond mandate or capacity of public health
	It was not helpful to have different terminology (i.e., ‘priority populations’) than community partners
Implementation by PHUs	Little conceptual clarity by policymakers themselves led to poorly defined mandate
	There were various interpretations of PPM actions and outcomes as these were not clearly linked or laid out
	Led to too much focus on identification of priority populations versus action on HE/SDOH

### Factor 1: Introducing new terminology (OPHS Policy Attribute)

The new term ‘priority populations’ caused confusion and inaction but also provided an opportunity to discuss health equity action in different ways.

#### Facilitator(s)

The introduction of new terminology opened up discussion on HE/SDOH practice in Ontario. Individual PHUs were able to “create a new concept and attach our own definition to [the term priority populations].” The new term ‘priority populations’ was seen as more proactive and actionable language than other terms such as ‘disadvantaged’, ‘marginalized’ or ‘vulnerable’ that are commonly perceived as synonymous. The term ‘priority populations’ (along with its description in the OPHS) was seen as “focussed on what can we do about people who experience health inequities rather than just sticking to the existence of health inequities.”

In addition, some participants discussed how the new term ‘priority population’ was value-neutral as compared to the terms ‘disadvantaged’, ‘marginalized’ or ‘vulnerable’. Participants saw the opportunity for the new language to be empowering for the populations identified as “priority” as the terms ‘disadvantaged’, ‘marginalized’ or ‘vulnerable’ do not acknowledge inherently the systemic issues (such as racism, ableism, sexism, etc.) that may cause disadvantage, and are consequently value-laden. Participants pointed out how this language, by identifying populations as priorities, transfers onus to the public health sector in terms of ensuring healthy populations. This language also implies that these populations are at a disadvantage because appropriate services are not available to them (i.e., they are a ‘priority’ through no fault of their own and this terminology represents that value-neutral stance).

#### Barrier(s)

Introducing a new term presented several challenges. Some found the term confusing and there was a sense that “people didn't grasp the definition of priority population[s] the same way” and it was “a challenge for some health units to get their heads around it.” A participant observed that “you can get so caught up in the semantics of it [what priority populations means]… [that] you can become paralysed with inaction.” It was perceived that action on health equity was delayed as practitioners focused on what ‘priority populations’ meant. Another issue that arose with the term ‘priority populations’ is that “priority suggests a ranking” and this issue of wanting to rank priority populations presented a barrier to action. In some cases, wanting to rank or prioritize populations led to a much longer process of identification of priority populations. According to one participant, “it can be challenging if there’s competing interests and it may be deemed that one population that’s chosen is in conflict with different programs, or the community, who’ve already chosen something else, so [the decision-making process] needs to be discussed.”

Lastly, there were those who felt that the ‘priority populations’ term itself, specifically the intentionally value-neutral nature of the term was a barrier to health equity action. Some felt that the term was too far removed from the roots of health equity work and was “not a useful term to address what it was meant to address, which is the social determinants of health.” Many felt that the language of the OPHS as a whole, including the ‘priority populations’ term, should more specifically “refer to those who may be at greater risk of health inequities due to modifiable social or economic factors.”

Finally, in spite of the incorporation of health equity into the public health mandate through the PPM, some practitioners felt that it was still challenging to “be able to defend it with the Board of Health (BOH) or with Council [who still think] ‘why is health encroaching on other people’s responsibilities’ and ‘if there’s limited funds, why go broader’?” This observation was linked to the OPHS “not going far enough” in addressing health equity. Respondents felt that if the PPM had been more strongly worded, BOH, which govern each health unit, would be more likely to accept upstream work.

### Factor 2: Allowing flexibility in implementation (OPHS Policy Attribute)

Several policymakers who were involved in the development of the OPHS highlighted that they intended the updated standards to be more flexible. One participant said, “what we were trying to do was give each health unit the flexibility of addressing the health needs, as [identified] by epidemiology, of their communities.” Since the priority populations for each health unit and program were not specified, identification of priority populations and subsequent action was left to individual health units.

#### Facilitator(s)

Some participants felt that the OPHS allowed flexibility in implementation as it outlined overall outcomes and general actions as opposed to specific outputs and deliverables that needed to be applied across the board. The idea was that the PPM would allow practitioners to “tailor some of their efforts.”

One participant provided an example of a prescriptive mandate, “you're told you must deliver this program to all women within 24 hours of when they have a baby,”and noted that in contrast, the PPM “was meant to give people a way to say ‘actually I am allowed to work specifically with groups I know are at risk and I can do something about. I don't have to do everything to all people’.” The PPM gave PHUs “an opportunity for some flexibility within the standards so that they could comfortably have room to do both more generic population level interventions and more specific interventions.”

In this way, the OPHS facilitated HE/SDOH practice by giving local PHUs autonomy in interpreting the PPM to fit their local population (although some thought the ‘priority populations’ term could have been better defined). The flexibility in the PPM allowed practitioners to assess situations using “local data and expertise” and to respond to the needs of the community. For instance, when PHUs “had groups they knew needed additional support in the community” the PPM was permissive enough that they could “have a public health intervention with those groups.”

#### Barrier(s)

On the other hand, flexibility, particularly the lack of explicit direction on how to identify priority populations, and ensure that programs and services meet their needs, was also identified as a barrier to action on HE/SDOH. Some practitioners wanted policymakers to “frame the requirements in such a way that [addressing health inequities] could have been much more explicit.”

One participant said, “after years and decades of trying to address health inequities… [we were] looking for a systematic set of solutions that are grounded in the requirements, both at the population health assessment level, but also program evaluations and the whole continuum of techniques that would provide feedback around how well we are doing in terms of identifying and meeting or mitigating health inequities… and the current OPHS doesn't go far enough.”

Some felt that identifying and addressing the needs of priority populations “has to be a top down mandate …for everybody to make it a priority to look at this work.” There were concerns that the flexibility in the OPHS allowed some BOH to implement the PPM in a way that would not result in meaningful impact on health equity. Participants thought that the provincial health government, the MOHLTC, needed to have “a stronger mandate… related to addressing first and foremost priority populations.”

Participants felt that without more explicit direction and accountability, the PPM may not be interpreted or implemented to address health equity, “if I've learned anything ‘post’ the OPHS it is that despite our best efforts to have fairly clear cut mandate, Boards of Health will, or staff will, interpret whatever to meet their own ends, even if you have a more well-defined and circumscribed definition, staff will use it or not as the circumstances and as their own preferences dictate.” There was concern that because the OPHS was “flexible”, the PHUs would not address HE/SDOH “unless they are told to do it.”

### Factor 3: Ensuring evidence-informed decision-making (EIDM) (OPHS Policy Attribute)

An important attribute of the OPHS was that needs-based service delivery must include surveillance, epidemiological or other research evidence to support delivery of programs and services [6].

#### Facilitator(s)

The EIDM attribute of the OPHS was considered to facilitate action on HE/SDOH by promoting objective conclusions that tended to be more favourably received by program funders and decision makers. The OPHS states, "priority populations are identified by surveillance, epidemiological, or other research studies and are those populations that are at risk and for which public health interventions may be reasonably considered to have a substantial impact at the population level" [6] (p.4). Participants highlighted that epidemiological evidence was “well-received” because it is important to be able to “defend your use of resources during this fiscally tight times.”

#### Barrier(s)

However, participants explained, “it’s actually very difficult to obtain disaggregated data at the regional or local level.” There are gaps in data on SDOH for specific populations and this is a barrier to action on HE/SDOH. Several respondents echoed that “without better data on risk factors, health behaviours and outcomes, we have very limited baseline data to identify priority populations, and also to monitor the outcomes of our interventions with these populations.” Practitioners often relied on their previous practice and experience to identify and take action on priority populations. One participant explained, “priority populations are being identified largely based on … our experience, the relationships we have in our community [and] through community consultations. It’s not as much based on the data, especially for the smaller priority populations, because we just don’t have the data.” However this approach can be problematic “if it looks like you are just picking a population to try and say[ing] I hope this works.” Practitioners struggled to provide epidemiological data that an SDOH was causing a particular negative health outcome.

### Factor 4: Different understandings of health equity (Health Sector Context)

In our report [9], we found that health equity was conceptualized in various ways, along a spectrum from a burden of disease approach on one end to a health equity approach on the other end. In addition, it has been shown that PHUs were working toward health equity in very different ways, from direct service provision to enhancement of socioeconomic conditions [11].

The health equity approach considers “health equity first, as inherent to public health practice and embedded in the social determinants of health, including service access” [9] (p. 123). The burden of disease approach considers “burden of disease first, as an objective starting point for the determination of priority populations.” The combined approach often sees “burden and determinants as overlapping or all part of a "mix of things" when it comes to considering priority populations” [ibid].

#### Facilitator(s)

The PPM recognizes both 'burden of disease first' and 'health equity first' approaches. The 'burden of disease first' approach is acknowledged through the statement that "priority populations are identified by surveillance, epidemiological, or other research studies and are those populations that are at risk and for which public health interventions may be reasonably considered to have a substantial impact at the population level" [6] (p.4). When describing the principle of need which underlies the definition of priority populations, the OPHS states that “need is established by assessing the distribution of determinants of health, health status, and incidence of disease and injury” [6] (p.19).

Some participants described moving forward on the 'burden of disease first' approach, which meant that “priority populations are identified through the routine analysis of health status data.” Other participants that described processes focused on 'healthy equity first', actively included priority populations in programs and services decisions to offset systemic inequities in health care and health outcomes or identified priority populations qualitatively, through community influence or practitioner experience. For instance, participants described how “our health unit is forming a workgroup to address SDOH” or through using “a health equity mapping checklist as a part of program planning.”

The 'health equity first' approach is acknowledged through the statement that BOH shall use “population health, determinants of health and health inequities information” [6] (p.24). The range of definitions in the PPM facilitates action on HE/SDOH by enabling practitioners to move forward *within their understanding* of how to address the issue of priority populations at the local level within separate jurisdictions and programs.

#### Barrier(s)

Since the PPM incorporated both 'burden of disease first' and 'health equity first' approaches to identify priority populations, practitioners have interpreted how to identify priority populations within their own epistemological paradigms, often leaning towards one approach over the other. Those who fell into the 'burden of disease first' approach felt it was clear that populations are “a priority based on the burden of illness or the increased risk of adverse health outcomes, so that’s where your research or surveillance will determine how much of a priority they will be.” Whereas those who fell into the 'health equity first' approach thought that “you use a robust understanding of determinants of health to identify, characterize priority populations” and that “we should always be using a health equity approach.”

Given these differences in opinion on how health equity should be addressed, practitioners may be “talking at cross-purposes” and consequently the PPM acted as a barrier to action on HE/SDOH. One participant described, *"*theoretically everyone agrees on SDOH but nothing happens."

Some practitioners felt that when priority populations were identified under the 'burden of disease first' interpretation, which primarily focused on “looking at cancer rates, looking at diabetes rates”, it took away from direct action on HE/SDOH. The ‘burden of disease first’ approach runs the risk of excluding criteria of disadvantage, and undermines health equity. This factor takes away from a values and experience based premise for focusing on HE/SDOH, which may not be construed as evidence-informed.

### Factor 5: Variability in existing partnerships (Health Sector Context)

The new PPM was introduced into an environment where PHUs had existing partnerships with communities, subpopulations and community organizations to take action on SDOH.

#### Facilitator(s)

Some respondents noted that the PPM facilitated HE/SDOH practice by supporting collaboration with other sectors. For some respondents, “knowing what public health’s role is and acting on it in terms of the social determinants of health” highlighted the need to “do what we can in terms of inter-sectoral partnerships and initiatives and advocacy… by looking upstream and addressing [health equity] outside the health sector, outside of just our direct programs and services, the interventions that we can have there.”

#### Barrier(s)

Participants noted that it was not helpful to have different terminology (i.e., priority populations) from their partners. Inconsistencies in terminology between sectors are a barrier “to doing this work properly” and “it would be helpful to use similar terminology…. to what our partners are saying and that would be [the terms] 'vulnerable populations' or 'disadvantaged populations'.” Sectors outside of public health “who are mandated to support populations at risk, they don’t use [the term] priority population, they’re more inclined to use wording such as vulnerable populations.”

A participant mentioned that we should know “what public health’s role is and acting on it in terms of the social determinants of health” but that there are opportunities “outside of the healthcare system, so doing what we can in terms of inter-sectoral partnerships and initiatives and advocacy [is important].” We need to look “at the bigger picture… looking upstream and addressing [priority populations] outside the health sector.” Participants identified the need to collaborate with other sectors to take action on HE/SDOH and not doing so could be a barrier.

### Factor 6: PHU implementation of the requirement to address PPM

The PPM was introduced into an environment where different PHUs were taking different types of action, and when they were mandated to meet the requirements of the OPHS, several PHUs needed to adjust current work or add new work to meet the mandate.

#### Facilitator(s)

Overall, the PPM served as a catalyst that increased “dialogue and pushed boundaries in terms of expectations”, increased service provision at the local level, made links to HE/SDOH and pushed PHUs to consider creative solutions. Participants described focusing their work on health equity “since 2008 [when the PPM was released], we’ve been acquiring the data that we need to understand our populations better and have been mapping data by health neighbourhoods.” The PPM spurred on new work and required that some PHUs develop additional materials (e.g. “priority population primer document”) to facilitate implementation of the mandate, “if we hadn’t done that [developed a primer document] it would have been very difficult for our planning staff to plan.”

The PPM also drew attention to the need for health equity capacity building within PHUs. One participant said, “we do not have capacity in the health department to easily collect new data on specific priority groups (they are difficult to find, methods may need to be non-traditional, and our resources are limited).” The participant went on to explain, “it’s not always clear whose responsibility it is to identify priority populations, especially if research and literature are being used versus surveillance and local data which is a more obvious [epidemiology] role. Lastly, once information is gathered, properly weighing the importance of the data, dealing with conflicting results/opinions, and dealing with the gaps in the data/knowledge can be challenging.”

To meet the requirements of the PPM, PHUs identified opportunities for PHU partnerships including sharing of resources, such as “if one health unit or if multiple health units come together and participate in a research project or look at the data analysis and identify three communities where indicators of chronic disease or hospitalizations consistently are more likely for people who are low-income or lower education.” The PPM facilitated existing action by enabling PHUs to “do what they need to” to address health inequities within their capacity and context, by incorporating their existing work into the OPHS framework.

#### Barrier(s)

The PPM required health units to “create their own health equity frameworks” to “actually operationalize what that process [of identifying priority populations and addressing SDOH] would look like,” because clear processes and outcomes were not laid out. As there was a lack of direction from the PPM, there were conflicting interpretations and actions that resulted. The PPM relied on the initiative of health units to do this work and as such, some PHUs were not able to do the additional work on priority populations.

Although some PHUs were able to do the additional work required to develop processes for working on priority populations in their jurisdiction, others faced multiple barriers due to limited capacity and resources, “some health units are smaller and to them this type of work is a pipe dream. [Identifying priority populations] depends where you live and how well you are funded, [which determines] whether there is a whole lot of work happening in this regard.”

Some participants thought that the PPM had too much focus and was “hung up” on identifying priority populations versus using the PPM as leverage to move forward and take action on health equity. However, as one practitioner involved in writing the PPM described, “I don’t think we meant to make it [identifying priority populations] a great work project for health units. I actually do think [the PPM] was just meant to ensure that people were thinking about their interventions, but to give that leverage to go back to if they wanted to do something with a specific group rather than having to do it across the board the same way to everybody.”

## Discussion

We explored how the PPM facilitated or hindered action on HE/SDOH. We found that the PPM both facilitated action on HE/SDOH (by increasing dialogue, raising awareness of capacity issues, organizing practice and directing resources as noted in Table 2) and inhibited action on HE/SDOH (by causing confusion, having little conceptual clarity, focusing on identification of priority populations and not action as noted in Table 3).

The PPM could have been strengthened to guide action on HE/SDOH if it introduced more clearly defined terminology. Although there are ways in which the PPM could drive action on health equity, it appears that overall the PPM was received by practitioners in a way that inhibited immediate action because they got caught up in understanding the term ‘priority populations’. Our study highlights that for health equity policies to be action-oriented, they need to be as clear and explicit as possible about terminology that is used. The National Collaborating Centre for the Determinants of Health (NCCDH) observed that the term ‘priority populations’ has the “risk that without specific inclusion of social justice values, the term can be interpreted too broadly, and be used to identify populations not experiencing disadvantages” [12] (p.4). The terminology is perceived by some as weakening the impact and link to health equity and resulted in confusion about whether the PPM was intended to address health equity at all.

The PPM could have been strengthened to guide action on HE/SDOH if it had a clear guiding health equity vision. The perceived weakening of the link to health equity by introducing the term ‘priority populations’ may have resulted from differences in opinion regarding the term ‘social justice’. As noted in our larger study report, six informants were in agreement that, “the term ‘social justice’ causes divide” [9] (p.25) and warned against its use. We heard that, “by introducing the term ‘social justice’you basically introduced a contentious lightening rod into the discussion” and “words like social justice or health equity could mean to someone [that] you are just trying to raise taxes to create more programs for the public sector” [9] (p.25). However, some participants wanted to push a social justice framework and said, “there is room for us to strengthen, disseminate and acknowledge a public health ethical framework that embeds social justice within it.” Therefore, the OPHS and PPM were perceived to fall short of committing to a clear health equity vision for public health in the province. This inhibited public health action as participants felt that a stronger top-down mandate was necessary to push work on HE/SDOH further and would solidify support from BOH in doing this work.

Finally, the PPM could also have been strengthened to guide action on HE/SDOH if it was able to unite different PHU approaches to addressing health equity. One of the reasons writing an overarching OPHS and PPM may have been difficult is because of the wide range of ways in which health equity has been operationalized in the province. Raphael and Brassolotto [11] discussed three differing approaches to addressing SDOH, 1) service delivery, 2) intersectoral and community, and 3) structural [11]. Pinto et al. [3] identified that the recognition of “how equity is conceptualized” would assist in developing “more explicit, action-oriented and concrete steps” to address health equity (p.8). At the same time, the intention of the OPHS to be flexible and not prescriptive is understandable, as it was meant for PHUs at the local level to adapt the mandate to their needs.

In spite of barriers described above, and without formal evaluation, many respondents felt that overall the PPM was a step in the right direction and that “we can safely say that in some cases work to address the needs of priority populations as prescribed by the mandate has resulted in an increase in direct service provision to priority populations.” However, the PPM seemed to only address health equity from a service-delivery perspective and did not acknowledge that some practitioners and PHUs had intersectoral and community or structural approaches to health equity [11]. To successfully reap the benefits of a flexible policy that can be adapted at the local level, it appears necessary to establish and communicate a complete vision of the ways in which health equity is operationalized.

### Limitations

Although we did not interview everyone involved in the development of the OPHS, we gained a range of perspectives from different key informants. Across 16 interviews, saturation was reached. However, it is possible that we may have missed information that could have been captured only from policymakers who did not participate. In the case of policymakers, participants were asked questions referring to the decisions that happened several years prior and consequently, there may be some recall bias. There may be some selection bias as only participants who were interested in providing their input may have agreed to be interviewed or completed the survey. By including qualitative responses from practitioners through semi-structured interviews as well as through open-ended survey questions, we were able to compare responses and ensure consistency in types of responses. Efforts were made to rigorously analyse the data (iterative coding of data and ensuring quotes were placed in context), and authors consistently engaged in reflecting on issues of personal bias.

This paper only focuses on health equity in the context of the PPM. Our analysis specifically assesses the intention of this particular policy, the OPHS, to improve health equity and the interpretation and application of this PPM within the local public health field toward health equity outcomes. This paper does not assess or explore the other policies, programs and efforts to address health equity. The findings from this paper contribute to the existing and emerging work being done in the area of priority populations and health equity, and should be contextualized accordingly.

## Conclusions

We found that the revised OPHS and the PPM facilitated action on HE/SDOH (by increasing dialogue, raising awareness of capacity issues, organizing practice and directing resources) but that on the whole, this objective could have been better met. The PPM could have been strengthened to guide action on health equity if 1) it introduced more clearly defined terminology, 2) had a clear guiding health equity vision and 3) was able to unite different PHU approaches to addressing health equity. For a policy to move forward action on health equity there needs to be clear terminology that explicitly articulates a health equity vision.

## Abbreviations

BOH: Boards of Health
EIDM: Evidence-Informed Decision Making
HE: Health Equity
HE/SDOH: Health Equity and Social Determinants of Health
KII: Key Informant Interviews
MHPSG: Mandatory Health Programs and Services Guidelines
NCCDH: National Collaborating Centre for Determinants of Health
OPHS: Ontario Public Health Standards
PHU: Public Health Unit
PPM: Priority Populations Mandate
SDOH: Social Determinants of Health

## Footnotes

1 This article uses the terms health equity/ social determinants of health (HE/SDOH) with the understanding that they are distinct concepts but that actions on the social determinants of health address underlying health inequities. A conscious decision was made to use health equity versus health inequality to acknowledge the health differences between populations that are unfair and avoidable [12].
